# A Comprehensive Clinical Perspective of Nasoalveolar Molding (NAM) for the Treatment of Cleft Lip and Palate patients

**DOI:** 10.4317/jced.62575

**Published:** 2025-05-01

**Authors:** Abdalmawla Alhussin Ali Ali

**Affiliations:** 1 Professor (assistant) in Department of Orthodontics, Faculty of Dentistry, Sirte University, Libya

## Abstract

**Background:**

Cleft lip and palate is widespread congenital abnormalities affecting the orofacial area. Nasoalveolar Molding (NAM) is a pre-surgical orthodontic method that has revolutionized the management of newborns with cleft lip and palate. Objectives: The purpose of this research are to present the steps of NAM appliance construction as well as to evaluate how presurgical nasoalveolar molding affected the long-term nasal, alveolar, and palate shape of infants with cleft lip and palate.

**Material and Methods:**

Individuals with complete unilateral cleft lip and palate are candidates for NAM treatment. Earlier intervention, within the initial weeks of life, is essential for attaining best outcomes. Patients must be assessed for their general health and appropriateness for the NAM management.

**Results:**

The improvement of nostril height was 0.8 mm after application of NAM appliance and after primary surgical repair by 0.7mm, whereas the nostril width reported decrease by 1.4 mm after utilization of NAM and by 1 mm after lip repair. Columella-nasal base angle increase from 51.8 º to 90.1 º after application of NAM appliance and primary surgical repair. There were reduction of alveolar gap width by 7.6 mm and 9.3 mm after application of NAM appliance and primary surgical repair respectively.

**Conclusions:**

Nasoalveolar molding is effective pre-operative treatment for newborns with cleft lip and palate in comparison to their birth status. The advantages of this advanced approach to cleft treatment will be further increased by continuous research and improvement of NAM procedures, as well as a strong focus on interdisciplinary collaboration.

** Key words:**Nasoalveolar molding for cleft lip and palate, Craniofacial anomalies, Pediatric dentistry, Infant Orthopedics, Pre-surgical orthodontics, Quality of life.

## Introduction

Cleft lip and palate (CLP) represents the most frequent congenital craniofacial defects, occurring in approximately 1 in 700 live births (1). These defects have substantially affected an infant’s speech, appearance, eating, and psychological development (2). This complex challenges require interdisciplinary treatment strategies, including pre-surgical orthopedic therapies. The surgeons and orthodontists began investigating non-surgical methods for assisting individuals with cleft lip and palate (3).

The most common cause of disturbances in maxillary growth of lip and palate children, particularly maxillary retrusion is the surgical repair procedure (4-6) and because of the extensive scar tissue that results from the soft tissues deteriorating after surgery (7). Therefore, minimizing scar tissue would be possible by decreasing the palatal gap in advance of surgery and choosing the appropriate reconstructive approach (8,9).

The development and enhancement of Nasoalveolar Molding (NAM) as a pre-operative therapy option were made possible by the important contributions of Millard (10-12).

The NAM appliances commonly begun in the first few weeks of infancy, the tissue plasticity which influencing the face growth and development in the early postnatal period. During this stage, infant tissues—especially bone and cartilage—show an extensive amount of plasticity (10). NAM necessitates good coordination between the parents, orthodontist, and surgical team. The NAM approach has achieved widespread recognition owing to its efficacy in improve nasal symmetry and reduce the space between the cleft segments, NAM uses mild, continuous pressure that guide their growth (13). In addition to that, NAM promotes more harmonious and aesthetically surgical outcomes by maximizing tissue alignment and decreasing the severity of clefts. Moreover, NAM can help with breastfeeding and bottle-feeding by enhancing the form and function of the oral cavity, which will benefit the nutrition and development of the baby (14). The application nasoalveolar molding decrease the soft tissue revision operations (15) and reducing the requirement for additional alveolar bone grafting (16). Furthermore, NAM can help affected individuals to achieve improved quality of lives in the future by addressing cleft-related concerns early in life (17). Despite its beneficial outcomes, NAM has disadvantages and drawbacks. The variables that must be considered which include the possible problems, possibility of relapse, the challenge of attaining ideal symmetry, related costs, patient compliance (18). The majority of NAM researches focused on improving nasal and alveolar deformities. Sasaki *et al*. (19) solely investigated the decrease in palatal cleft gap in individuals with unilateral cleft palates in one NAM research. They found that the NAM approach decreased the alveolar as well as palatal gap widths more than the achieved by Hotz plate. They highlighted the significance of palatal cleft size because they noticed a correlation between of the palatal cleft width and the surgical result of cheiloplasty (19).

The objective of the current study was to assess and compare the effectiveness of NAM intraorally, particularly on the palatal cleft region of patients, because there is a deficiency of statistical data about the effects of NAM therapy on this region.

Material and Mehods

**Figure 1:**
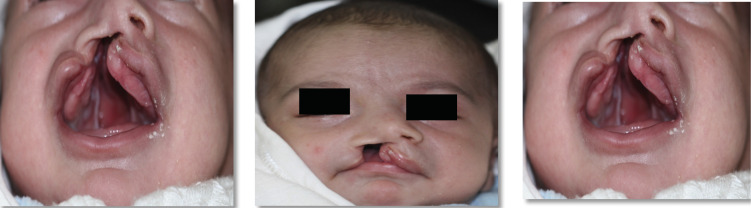
8 day old male was referred to orthodontic department from plastic surgeon for NAM. Birth weight 2.35 kg and medical history was unremarkable. On examination, nonsyndromic right side UCLP.

**Figure 2:**
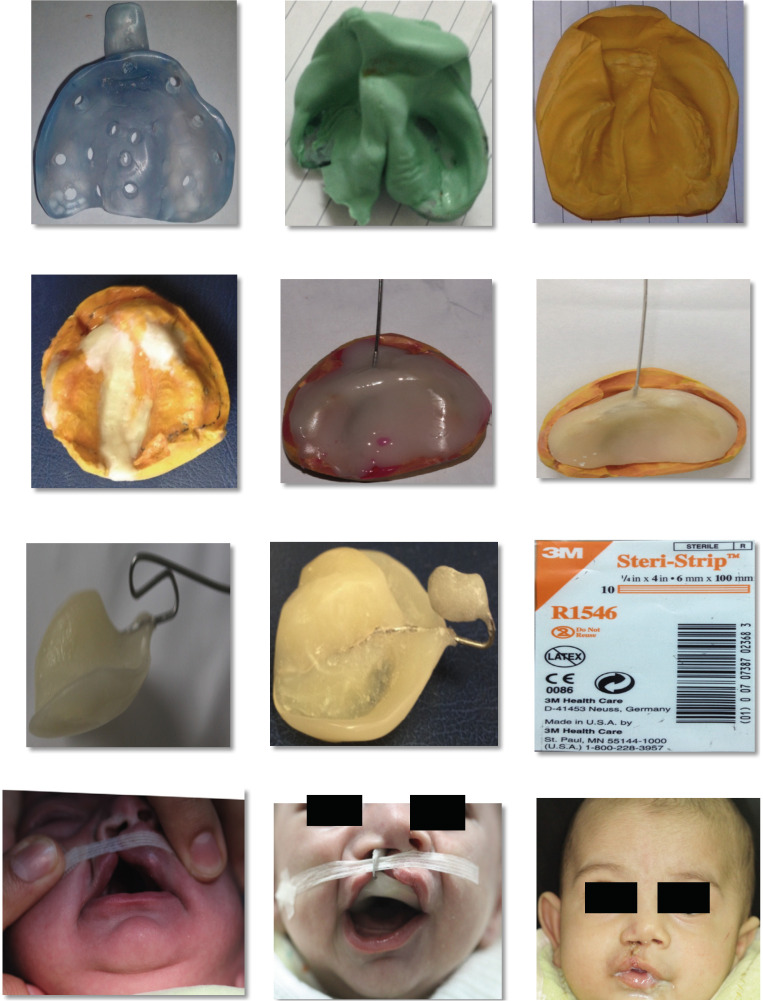
Treatment sequence with the nasoalveolar molding (NAM).

This study provides a comprehensive review of the NAM in our practice, involving patient selection, manufacturing and insertion procedures, clinical results, and factors for effective implementation. A collection of case reports illustrates the efficacy of NAM in attaining excellent surgical outcomes as shown in Figure 1. In the following are the therapeutic protocol for NAM as shown in Figure 2, which includes.

Comprehensive clinical assessment of individual: a complete examination of the infant’s cleft feature, feeding challenges, and general health state is performed.

Eligibility Conditions: NAM is usually considered for infants with a variety of cleft types, including as unilateral and bilateral cleft lip and palate. However, depending on the unique characteristics of each patient, the precise criteria for inclusion and exclusion may change.

Parental Counseling: Parents get thorough counseling on the NAM process, including its advantages, possible risks, and the degree of parental engagement needed.

Making an impression: Start utilizing heavy-bodied silicone impression materials to take a precise impression of the baby’s cleft region. To avoid obstructing the airway, the baby remains supine. For obtaining a precise impression of the alveolar segments, palate, and cleft lip, a customized tray has been prepared up. Careful consideration will be taken to recording the fine characteristics of the surrounding tissues and cleft borders.

Appliance Design and Fabrication: The impression is poured into a dental stone model to create a working cast, providing a three-dimensional representation of the infant’s oral structures. The models are carefully analyzed to assess cleft severity, identify areas for improvement, and plan the design of the NAM plate. An acrylic molding plate is fabricated over the cast, extending to cover the palatal shelves and the alveolar ridges. The orthodontist designs an acrylic molding plate based on the model analysis and treatment goals. This plate extend to cover the palatal shelves and the alveolar ridges.

Incorporating Nasal Stents: In order to provide progressive developing and eliminate nasal abnormalities including columellar shortening and alar collapse, nasal stents, which usually composed of acrylic or other appropriate materials, are integrated into the plate. To ensure optimal comfort, the appliance has been polished and smoothed.

Insertion the NAM Appliance: The NAM is gently placed in the baby’s mouth and secured with tape or adhesive. The parents received comprehensive instructions on how to maintain and take care of NAM. To guarantee an appropriate fit and convenience, the first adjustments typically occur just after the plate is inserted. Weekly visits to assess progress and make required modifications. The orthodontist to assess tissue response, modify nasal stents, and improve plate fit carries these out. The NAM appliance is regularly adjusted to progressively shape the nasal cartilages and alveolar segments. The NAM adjustments by Adding or removing acrylic material for controlling the tissue development. Parents receive comprehensive education about NAM. The NAM is worn continuously except during feeding and cleaning. The effectiveness of NAM treatment depends on the active participation of parents. It is advised that parents keep monitors on their baby’s comfort, follow the treatment plan, and communicate about any concerns immediately.

The standardized parameters measurements were made on the cleft dimensions both before and after treatment with NAM and surgery at T1 (Initial visit), T2 (After nasoalveolar molding), and T3 (Month after primary surgical repair), which include:

1-Nostril height: Highest point of nostril perpendicular to reference line in each nostril.

2- Nostril width: Distance from point farthest right to point farthest left of nostril on each side.

3- Columella-nasal base angle: A line bisecting columella from tip of nose to reference line, and angle was measured from affected nostril.

4- Alveolar gap width: Distance between segments of alveolar ridge using caliper.

Ethical approval: This study followed the principles of the Helsinki Declaration. Sirte University’s Research Ethics Committee approved the application (April 2024/Reference Code: 01).

## Results

**Figure 3:**
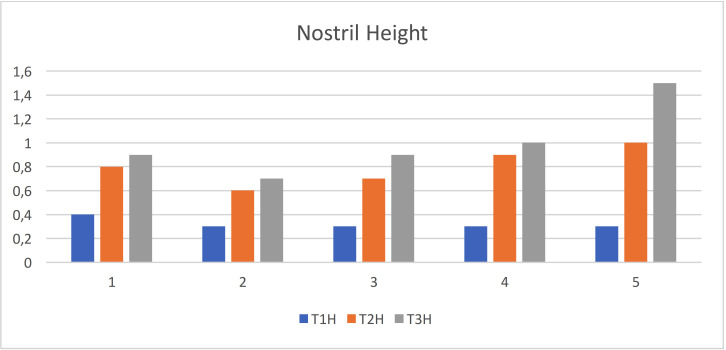
Statistical analyses of the quantity changes of nostril height.

Nostril height: The mean value and standard deviation of the quantity changes of nostril height at different interval time T1 (initial visit), T2 (after nasoalveolar molding), and T3 (month after primary surgical repair) are shown in the  Table 1 and Figure 3. The results illustrated that there were improvement in the nasal asymmetry where there were increase in the height of nostril after application of NAM appliance by 0.5 mm and after primary surgical repair by 0.7mm.

**Figure 4:**
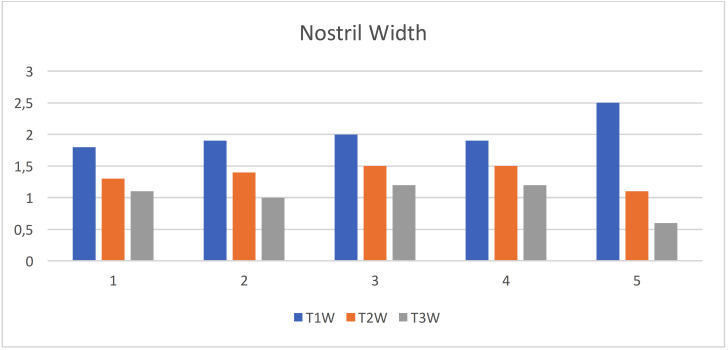
Statistical analyses of the quantity changes of nostril width.

Nostril width: The mean value and standard deviation of the quantity changes of Nostril width at different interval time T1 (initial visit), T2 (after nasoalveolar molding), and T3 (month after primary surgical repair) are shown in the  Table 2 and Figure 4. The results illustrated that there were decrease in the width of nostril, which reduced after usage of NAM appliance by 0.7 mm and after primary surgical repair by 1 mm. These reductions reflect the overall improvement in the columellar shortening and alar collapse resulting in improved function of the nasal airways.

**Figure 5:**
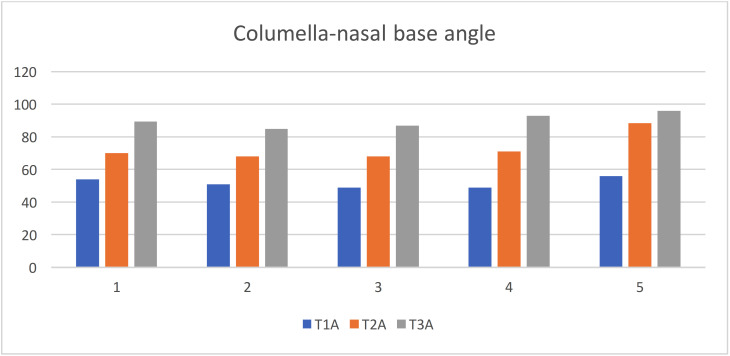
Statistical analyses of the quantity changes of columella-nasal base angle.

Columella-nasal base angle: The mean value and standard deviation of the quantity changes of columella-nasal base angle at different interval time T1 (initial visit), T2 (after nasoalveolar molding), and T3 (month after primary surgical repair) are shown in the  Table 3 and Figure 5. The results illustrated that there were improvement in the nasal abnormalities where there were increase in the columella-nasal base angle after application of NAM appliance by 21.3º and after primary surgical repair by 38.3º.

**Figure 6:**
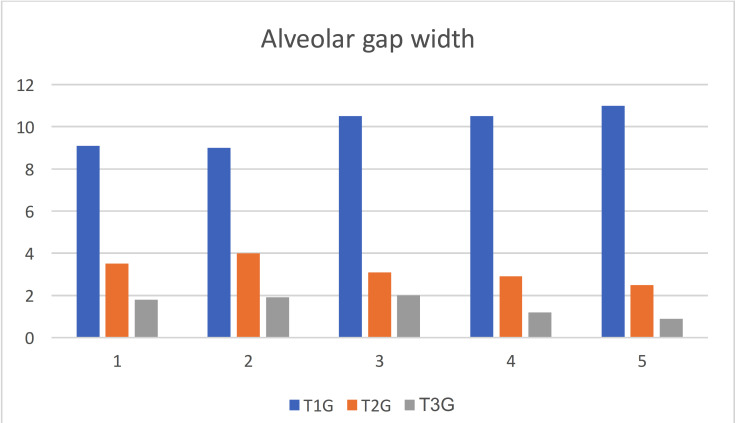
Statistical analyses of the quantity changes of alveolar gap width.

Alveolar gap width: The mean value and standard deviation of the quantity changes of alveolar gap width at different interval time T1 (initial visit), T2 (after nasoalveolar molding), and T3 (month after primary surgical repair) are shown in the  Table 4 and Figure 6. The results illustrated that there were decrease in the width of alveolar gap, which reduced after usage of NAM appliance by 7.6 mm and after primary surgical repair by 9.3 mm. These reductions reflect the dramatic improvement in the shape and functionality of the oral cavity and lowering the risk of aspiration and feeding challenges. In addition to that, it provides aligned alveolar segments, less severe cleft, reduced further surgical operations required, and an important improvement in aesthetic results.

## Discussion

Infants with cleft lip and palate treated by combination of orthopaedics and surgery. All cases began treatment by NAM appliances within the first week after birth. The individuals had a complete unilateral lip and palate clefts. The nasal alveolar molding was utilized prior to the lip repair surgery. Following orthopedic therapy, the clefts gap decreased as a result of the alveolar segments’ approximation with NAM. Contrary to what certain authors, the lip was surgically corrected in a single procedure due to the positive outcomes (20,21). The patient can benefit considerably from the application of NAM. According to several authors (19,22-26), the objectives of NAM are to restore symmetry to severely malformed nasal cartilages, project the collapsed nasal tip, elongate the columella nonsurgically, improve alveolar ridge alignment, and minimize the distance between the segments of the cleft lip.

The results of this study is supported by the fact that NAM may be used for all cleft abnormalities, including full clefts without an intact nasal floor (27).

Lip taping is one of several pre-operative patient orthopedics methods that have been documented. It considered as a supplemental approach to NAM treatment. Prior to the beginning cleft lip repair, this procedure mobilizes and approximates soft tissues of the lip, nose, and maxilla (28). For the orthodontist, correcting the cleft nasal deformities present a significant aesthetic challenges. When the gap in the alveolar cleft was decreased, the infant has utilized an established stent that is incorporated within the NAM appliance.

Subramanian *et al*. (23) modified the NAM technique by employing titanium molybdenum alloy wires (TMA) to create the nasal stent for cases with unilateral cleft palate. Because of the TMA wire’s increased resilience, activation may be done once every two weeks.

Matsuo *et al*. (29) found that cartilage alar is more pliable to the orthopedic appliances shortly after birth. this as a result of increase amounts of maternal estrogen in the child’s circulation and sialuronic acid in the nasal cartilage. The patients are required to benefit on the cartilage’s flexibility, which decreases after three months of age. In order to encourage permanent maintenance in its form (10), the authors state that pre-operative non-surgical corrections should start as soon as achievable (30,31).

Lip taping is an inexpensive and simple procedure. In our study, it was utilized as a supplementary treatment to NAM (10). It is administered to the lip across the cleft immediately following birth to remodel and approximate the alveolar arch. Additionally, the application of tape combined with elastic had been designed to help secure NAM on the infant’s face (28). Improved post-surgical outcomes appear to be mostly dependent on the NAM pre-operative therapy(21–24).

These results demonstrated in comparative researches and in this current study, where patients treated with NAM experienced improved functional and cosmetic outcomes (20). As previously stated, the literature provides a comprehensive description of early-cleft treatment utilizing NAM. The benefits of pre-operative orthopedics are obvious (32). Nonetheless, the most important factor, which contributed to the therapy successful, was parental cooperation. Because of the extreme deformity of the nose, lip, and maxillary arch, the delivery of infant with a cleft can be traumatic and challenge for the family (27).

## Conclusions

For infants with cleft lip and palate, nasoalveolar molding is an essential presurgical orthopedic procedure that greatly improves treatment results. In addition to enhancing nose symmetry and alveolar alignment, the NAM method makes following surgical treatments less complicated. Further investigation and development of NAM techniques will increase its efficacy and expand its applications in craniofacial abnormalities.

Recommendation:

For successful implementation of NAM appliances should consider the following points 

• Multidisciplinary team approach: Orthodontists, pediatricians, speech-language pathologists, plastic surgeons, and other cleft care team members must work together for effective implementation of NAM.

• Parental responsibility: The effectiveness of NAM treatment depends on the active involvement of parents.

• Continuous monitoring and adjustments: To maximize treatment results, careful observation and frequent NAM plate adjustments are essential.

• Managing possible complications: It is essential to take quick and efficient care of any issues such plate removal, mouth ulcers, and skin irritation.

## Figures and Tables

**Table 1:** Statistical analyses of the quantity changes of nostril height.

Cases	T1	T2	T3
1	0.4 mm	0.8 mm	0.9 mm
2	0.3 mm	0.6 mm	0.7 mm
3	0.3 mm	0.7 mm	0.9 mm
4	0.3 mm	0.9 mm	1 mm
5	0.3 mm	1 mm	1.5 mm
M and SD	0.32 mm ± 0.04	0.8 mm ± 0.16	1 mm ± 0.3

**Table 2:** Statistical analyses of the quantity changes of nostril width.

	T1	T2	T3
1	1.8 mm	1.3 mm	1.1 mm
2	1.9 mm	1.4 mm	1 mm
3	2 mm	1.5 mm	1.2 mm
4	1.9 mm	1.5 mm	1.2 mm
5	2.5 mm	1.1 mm	0.6 mm
M and SD	2.02 mm ± 0.28	1.36 mm ± 0.17	1.02 mm ± 0.25

**Table 3:** Statistical analyses of the quantity changes of columella-nasal base angle.

	T1	T2	T3
1	54 º	70 º	89.5 º
2	51 º	68 º	85 º
3	49 º	68 º	87 º
4	49 º	71 º	93 º
5	56 º	88.5 º	96 º
M and SD	51.8 º ± 3.11	73.1 º ± 8.71	90.1 º ± 4.44

**Table 4:** Statistical analyses of the quantity changes of alveolar gap width.

	T1	T2	T3
1	9.1 mm	3.5 mm	1.8 mm
2	9 mm	4 mm	1.9 mm
3	10.5 mm	3.1 mm	2 mm
4	10.5 mm	2.9 mm	1.2 mm
5	11 mm	2.5 mm	0.9 mm
M and SD	10.02 mm ± 0.91	3.2 mm ± 0.57	1.56 mm ± 0.48

## Data Availability

The datasets used and/or analyzed during the current study are available from the corresponding author.
